# Inbreeding reduces fitness in spatially structured populations of a threatened rattlesnake

**DOI:** 10.1073/pnas.2501745122

**Published:** 2025-08-18

**Authors:** Meaghan I. Clark, Eric T. Hileman, Jennifer A. Moore, Lisa J. Faust, Randall E. Junge, Brendan N. Reid, Danielle R. Bradke, Gideon S. Bradburd, Sarah W. Fitzpatrick

**Affiliations:** ^a^Department of Integrative Biology, Michigan State University, East Lansing, MI 48824; ^b^Ecology, Evolution, and Behavior Program, Michigan State University, East Lansing, MI 48824; ^c^W.K. Kellogg Biological Station, Michigan State University, Hickory Corners, MI 49060; ^d^Department of Ecology and Evolutionary Biology, University of California, Santa Cruz, CA 95060; ^e^Wildlife and Fisheries Resources Program, School of Natural Resources and the Environment, West Virginia University, Morgantown, WV 26506; ^f^Biology Department, Grand Valley State University, Allendale, MI 49401; ^g^Alexander Center for Applied Population Biology, Lincoln Park Zoo, Chicago, IL 60614; ^h^Department of Conservation Medicine, Columbus Zoo and Aquarium, Powell, OH 43065; ^i^Department of Ecology, Evolution, and Natural Resources, Rutgers University, New Brunswick, NJ 08901; ^j^Department of Biology, Université de Namur, Namur 5000, Belgium; ^k^Warnell School of Forestry and Natural Resources, University of Georgia, Athens, GA 30602; ^l^Department of Ecology and Evolutionary Biology, University of Michigan, Ann Arbor, MI 48109

**Keywords:** inbreeding depression, population structure, inbreeding, pedigrees

## Abstract

Small and fragmented populations are at high risk of local extinction, in part because of elevated inbreeding and subsequent inbreeding depression. A major conservation priority is to identify the mechanisms and extent of inbreeding depression in small populations. The eastern massasauga (*Sistrurus catenatus*) rattlesnake is listed as Federally Threatened in the United States, having experienced significant habitat fragmentation and concomitant population declines over the past 200 years. Here, we use long-term monitoring of two wild populations of eastern massasaugas in Michigan to estimate the extent of inbreeding in each population, identify mechanisms that generate inbreeding, and test for the impact of inbreeding on fitness. Using targeted genomic data and spatial coordinates of capture locations from over 1000 individuals, we find evidence of inbreeding and link inbreeding to spatial kinship structure within populations, possibly driven by limited dispersal. We reconstruct multigenerational pedigrees for each population to measure reproductive output and use long-term capture–recapture data to estimate individual survival (i.e., the two major components of fitness). We find evidence of inbreeding depression in both fitness metrics. The 5% most inbred individuals are 13.5% less likely to have any surviving offspring and have 11.6% lower annual survival compared to all less inbred individuals. By combining genomics and long-term monitoring data, we are able to link the life history of eastern massasaugas to inbreeding and detect relationships between fitness and inbreeding. These insights provide important conservation context for future management and for understanding how spatial structure can generate inbreeding depression even at fine spatial scales.

## Significance

Inbreeding depression—decreased fitness caused by parental relatedness—can reduce population viability and thus is a frequent focus in conservation. However, such effects are difficult to detect in wild populations because it is challenging to accurately measure fitness. In two small populations of imperiled rattlesnakes, we combined long-term monitoring efforts with genomic data to reveal that inbreeding decreases fitness via reproduction and survival—a clear sign of inbreeding depression. Populations also show fine-scale spatial clustering of relatives, likely due to limited dispersal. This pattern contributes to inbreeding and, in turn, inbreeding depression. Our work demonstrates the consequences of habitat fragmentation and the importance of long-term studies to understand the intersection of life history and demography and its implications for conservation.

Anthropogenic land use changes have dramatically altered the size and distribution of natural populations (1). In many species, contiguous ranges have been divided, and populations are restricted to isolated habitat fragments. Small and isolated populations are inherently more vulnerable to extinction than large, connected ones due to demographic and environmental stochasticity, reduced adaptive genetic variation, and inbreeding depression (2–4). Inbreeding depression, here defined as a reduction in fitness due to individual inbreeding or shared ancestry of an individual’s parents (5), can ultimately reduce population viability (3), increasing the probability of extinction. Identifying how and why inbreeding depression occurs in small populations and by how much fitness is reduced is therefore essential for the conservation of endangered species (6–8).

Fitness reduction can be the outcome of two distinct processes: genetic drift, which can drive deleterious alleles to higher frequency in a population, and nonrandom mating, which can increase individual inbreeding (3, 6). Small populations are at greater risk of a reduction in fitness due to genetic drift because the magnitude of changes in deleterious allele frequencies is inversely correlated with effective population size (4, 9–11). Nonrandom mating, e.g., between relatives within a population, can generate pedigree reticulations and concomitant increases in the proportion of an individual’s genome that is identical-by-descent to itself (i.e., coinherited from the same recent ancestor). Fitness is reduced in inbred individuals via an increase in expression of deleterious recessive alleles (dominance coefficient < 0.5) in homozygous genotypes and/or a reduction in heterozygosity at loci that convey a fitness advantage in heterozygous genotypes (overdominance) (12). Inbreeding due to nonrandom mating can be generated by a variety of behaviors and life history traits that generate substructure within populations, including the mating system (13), the age structure of reproducing individuals (14), and limited dispersal distances (15).

Despite the importance of understanding and quantifying inbreeding depression and its ubiquity under experimental conditions (12, 16), inbreeding depression remains difficult to detect in natural populations, especially in those not easily monitored due to low encounter probabilities (3, 17–19). Inbreeding depression driven by nonrandom mating within wild populations is typically assessed by testing for a relationship between individual inbreeding and an individual fitness component (or proxy) using regression models (18). Although genomic data have allowed for accurate estimates of individual inbreeding in natural populations, measuring fitness directly remains difficult for most species (5, 20, but see 21). Inbreeding depression varies in its impact on different components of fitness (22, 23), and commonly used fitness proxies, like body condition, are often not strongly correlated with fitness (24), potentially leading to high false negative rates in tests for inbreeding depression in the wild. Here, we demonstrate the value of long-term population monitoring by testing for inbreeding depression using estimates of survival and reproductive success within wild populations of the elusive eastern massasauga (*Sistrurus catenatus*).

The eastern massasauga is a wetland-specialist rattlesnake that has experienced significant habitat fragmentation over the past 200 years (25). It is federally listed as threatened under the United States Endangered Species Act and the Canadian Species at Risk Act. Although there is evidence that eastern massasaugas historically existed in fragmented populations due to their association with naturally patchy wetland habitats, populations have experienced recent declines and extinctions associated with human land-use changes (26), and levels of natural connectivity have almost certainly been reduced. Contemporary populations of eastern massasaugas have small effective population sizes (27–29) and are vulnerable to habitat fragmentation, with agriculture, roads, and other human-modified habitats known to act as barriers to dispersal (30–33). Thus far, studies examining inbreeding have found no evidence of inbreeding depression in eastern massasaugas (29, 34), but these studies used body condition as a fitness proxy, as measuring fitness in this species is difficult without extensive long-term monitoring and genetic parentage information.

In this study, we merge over a decade of population monitoring with genetic pedigree reconstruction to quantify inbreeding in two populations of eastern massasaugas and test whether these small populations experience inbreeding depression. We 1) characterize the extent of individual inbreeding using genetic data, 2) analyze spatial structure that reinforces inbreeding, and 3) test for inbreeding depression using survival estimates from capture–recapture data and reproductive output measured from reconstructed wild pedigrees. We find that inbreeding impacts both survival and reproductive success in these populations and that the geographic scale of dispersal and mating within populations may contribute to the prevalence of inbreeding. Our results highlight how aspects of life history, such as dispersal limitation, may contribute to inbreeding and thereby its fitness consequences.

## Results

### Genotyping Individuals from >10 y of Capture–Recapture Monitoring.

We focused on inbreeding dynamics in two populations of eastern massasaugas in southwestern MI. We refer to these populations by their counties of origin: Barry and Cass. Field surveys at each site have been conducted since 2009 (Cass) or 2011 (Barry). Snakes were encountered within a small spatial extent [max. distance between snakes: 1.7 km (Cass) and 2 km (Barry)]. To estimate the extent of inbreeding and reconstruct pedigrees for each population, we used a RADseq + capture (i.e., RAPTURE) approach to target putatively neutral single-nucleotide polymorphisms (SNPs) known to be polymorphic in these populations (35). Initial quality filtering resulted in a dataset of 5,607 SNPs in 1,056 individuals at an average depth of coverage of 54X. After filtering for missing data and linkage, we retained a final dataset of 2,176 SNPs sequenced in 1,037 individuals across the two focal populations (Barry: *n* = 260, Cass: *n* = 777, 86 km apart). Contemporary effective population sizes, estimated using linkage disequilibrium (36, 37), were 48.82 (CI: 26.37 to 68.88) and 27.05 (CI: 21.87 to 31.63) at Barry and Cass Counties, respectively.

### Estimating Reproductive Output Through Pedigree Reconstruction.

**Fig. 1.**
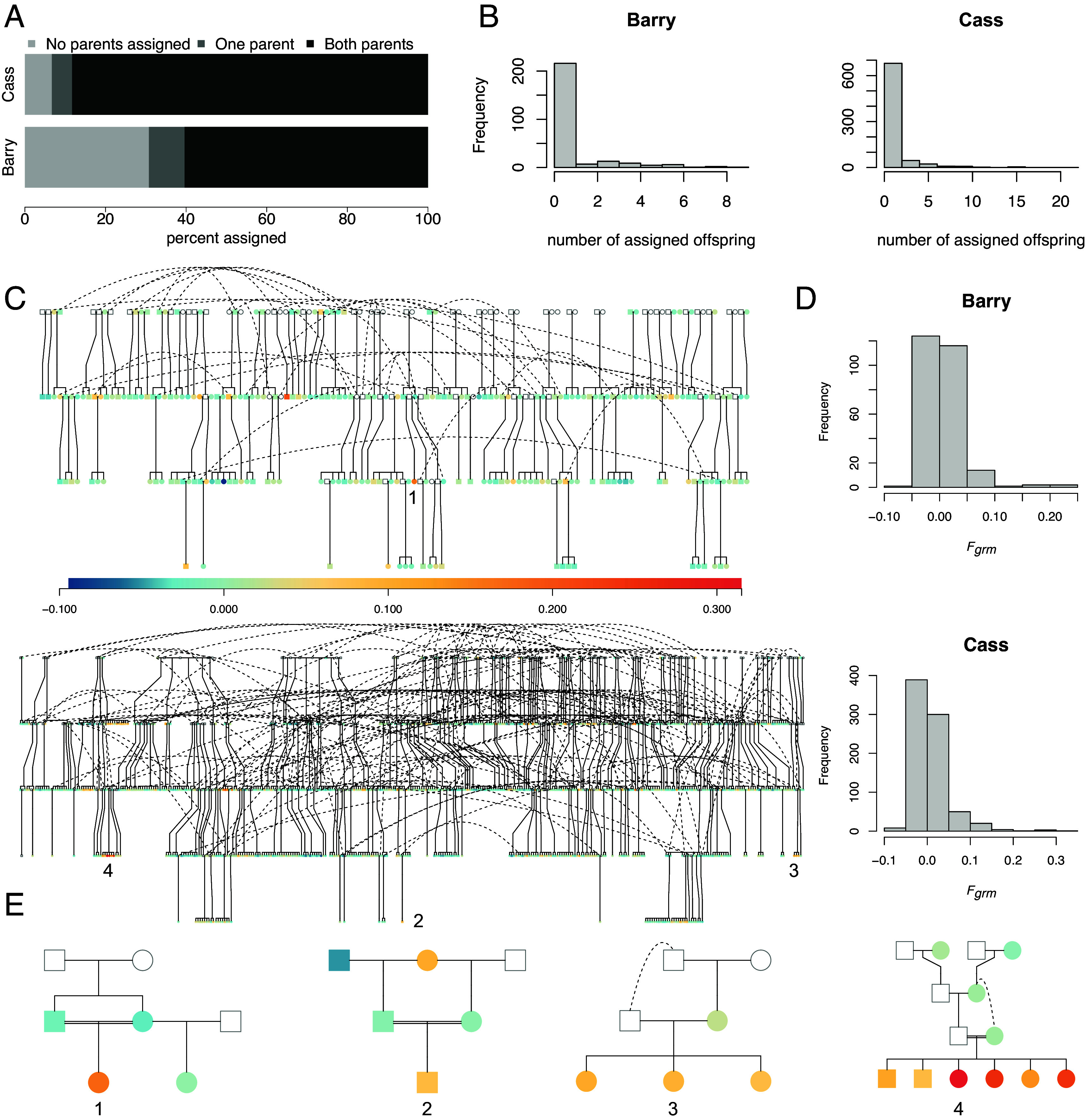
(*A*) Figure shows the proportion of individuals in Barry and Cass Counties who were assigned no, one, or two parents in pedigree. (*B*) Histograms show the number of assigned offspring for Barry and Cass Counties. (*C*) Reconstructed pedigrees of eastern massasaugas in Barry County (*Top*) and Cass County (*Bottom*), MI. Genotyped individuals are colored in accordance with *F_grm_*, and nongenotyped “dummy” individuals are outlined in gray. Pedigrees have been simplified for visualization, and some individuals appear multiple times. (*D*) Histograms show the distribution of inbreeding in Barry and Cass Counties. (*E*) Family pedigrees show instances of close-kin mating that resulted in offspring with elevated *F_grm_*. Numbers mark the location of focal individuals in both the family and population-wide pedigrees.

We reconstructed pedigrees separately for each population with the R package Sequoia (38), using SNPs with minor allele frequencies above 0.1 in the focal population, as loci with more common minor alleles provide greater information for pedigree reconstruction (Barry: 1,452 SNPs, Cass: 1,472 SNPs). We used a genotyping error rate of 0.00101 (CI: 0.000849 to 0.00117) based on base-pair differences between 30 individuals sequenced twice. Most individuals had parents assigned in the reconstructed pedigrees (Fig. 1, Barry County: 60.0% of 138 females and 119 males had both parents assigned, 9.0% had one parent assigned, 31.1% had no parents assigned, Cass County: 88.0% of 346 females and 278 males had both parents assigned, 5.0% had one parent assigned, 7.0% had no parents assigned). Parents were either genotyped individuals or “dummy” individuals, the latter of which are inferred to exist via pedigree relationships between relatives. At Cass and Barry Counties, respectively, 70.4 and 48.1% of individuals had genotyped dams, and 43.6 and 33.8% of individuals had genotyped sires. The discrepancy in parents assigned between populations reflects differences in population size and survey effort. Dams and sires had between one and nine pedigree offspring at Barry County and between one and 22 offspring at Cass County (Fig. 1). A total of 65.9% (*n* = 91) and 77.6% (*n* = 342) females and 75.6% (*n* = 90) and 80.0% (*n* = 267) of males at Barry and Cass Counties, respectively, did not have offspring assigned in the pedigree, suggesting substantial variation in reproductive success. These counts include both individuals for whom we have lifetime reproductive success and individuals who had limited reproductive opportunities due to their age (see Methods and Supplemental Methods). Pedigrees encompassed a maximum of three generations at Barry County and eight generations at Cass County.

Multiple lines of evidence suggest high confidence in inferred pedigree relationships. First, we included 265 snakes born in the lab to known dams (and subsequently released) (39, 40), and all but one of these relationships were correctly inferred in our pedigree reconstruction, with the exception of one lab-born snake who was not assigned parents. Second, we only allowed types of pairwise relationships (e.g., both parent and offspring genotyped; offspring is genotyped, parent is dummy) for which the assignment confidence probability for the pairing was ≥ 95% (*SI Appendix*, Tables S1 and S2). Third, to test whether we are undercounting offspring for snakes captured at the edge of the sampled area, we conducted linear regressions between number of offspring and geographic distance from the center of each site and found that distance from the center was not a significant predictor of number of offspring at either site [Cass: R^2^ = −0.000600, *F*(1,618) = 0.632, *P* = 0.427, Barry: R^2^ = −0.00356, *F*(1,254) = 0.0954, *P* = 0.758].

### Genomic and Pedigree Inbreeding in Eastern Massasauga Populations.

We estimated genomic inbreeding for individuals in each population separately using *F_grm_*, a measure of individual inbreeding (5). Many calculations of genomic inbreeding exist; here, we use *F_grm_* because it places more weight on minor allele homozygotes that are less likely to be observed by chance compared to major allele homozygotes and is commonly used in studies with similar SNP datasets (41, 42). Because *F_grm_* compares individual homozygosity to population allele frequencies, it is more sensitive to inbreeding from nonrandom mating than genetic drift. Values of *F_grm_* ranged from −0.0946 to 0.248 at Barry County and from −0.0724 to 0.315 at Cass County (Fig. 1*D*). Negative values represent an excess of heterozygosity, reflecting individuals that are more outbred than expected given population allele frequencies. At Cass and Barry Counties, 10.2 and 7.3% of individuals had *F_grm_* values greater than 0.05, respectively (Fig. 1*D*). One individual from Cass County had an extremely elevated *F_grm_* value (0.88), reflecting an excess of minor allele homozygous genotypes. We removed this individual from downstream analyses as a likely migrant.

We also used the inferred pedigree to calculate pedigree inbreeding coefficients (measures of the relatedness of inferred parents in the pedigree). Pedigree inbreeding ranged from 0 to 0.25 in both populations. At Barry County, one out of 37 unique pairs of genotyped parents were full siblings. At Cass County, seven of 99 unique pairs of genotyped parents were half-siblings. The offspring of the most related pairs of parents in the pedigree also had elevated *F_grm_* [*r*(1034) = 0.394, *P* < 0.001], (*SI Appendix*, Fig. S1).

### Spatial Genetic Structure Detected Within Each Population.

**Fig. 2.**
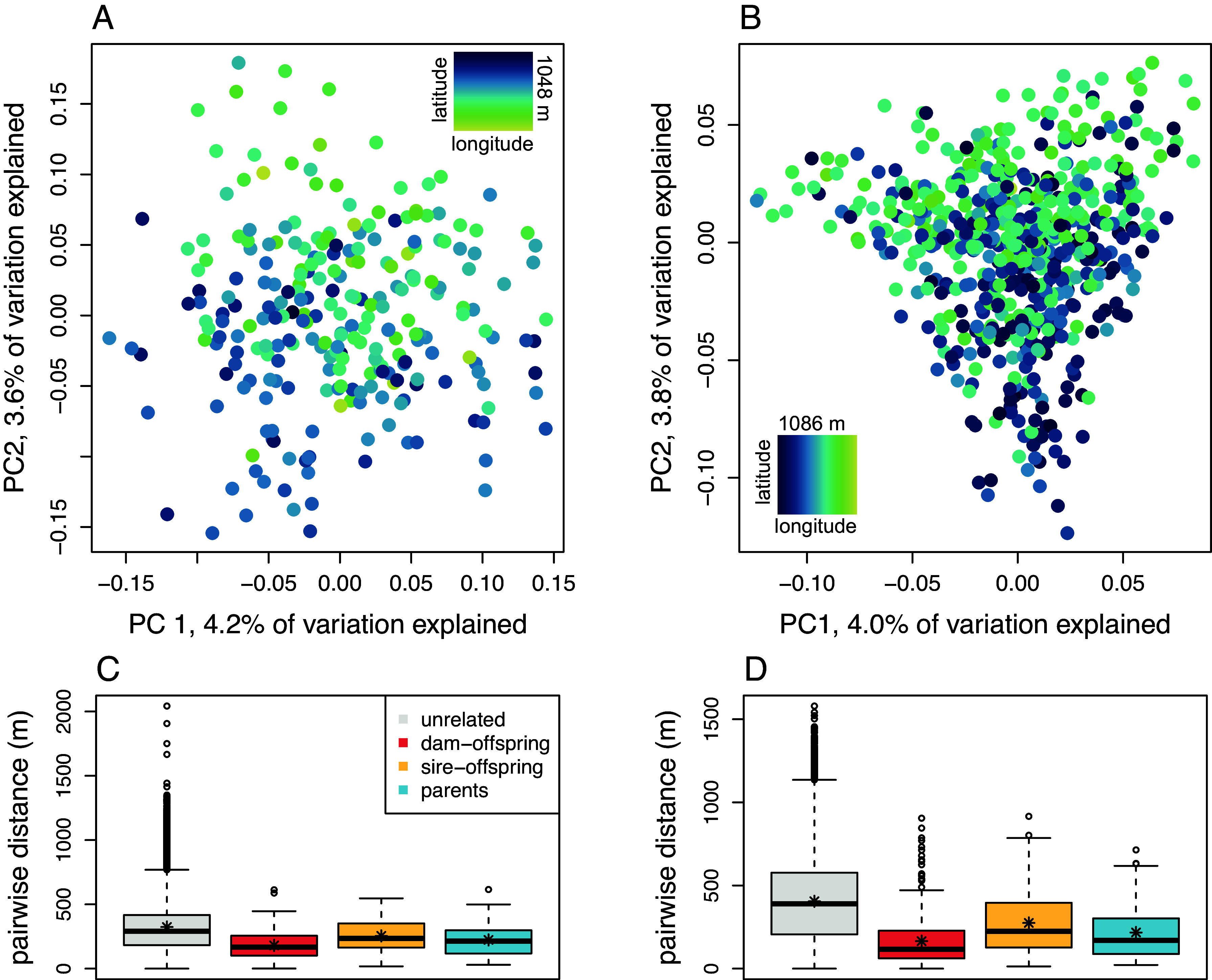
*Top* plots show genetic principal component analyses for eastern massasaugas at Barry (*A*) and Cass (*B*) County sites. Points are colored according to an individual’s centroid latitude at Barry or longitude at Cass, visualizing the main spatial axis of genetic variation within each site. The maximum distance between individual centroids along a latitudinal axis at Barry County was 1,048 m, and the maximum distance between individual centroids along a longitudinal axis at Cass County was 1,086 m. *Bottom* plots show distributions of pairwise distances in meters between unrelated pairs without offspring (gray), dam–offspring pairs (orange), sire–offspring pairs (yellow), and parents with offspring in the pedigree (blue) for Barry (*C*) and Cass (*D*) County sites. The bar and star on each boxplot represent the median and mean of pairwise distances, respectively. All comparisons between dam–offspring, sire–offspring, and parent pairwise distance distributions and unrelated distance distributions are significant (*P* < 0.05) using Kolmogorov–Smirnov tests with Bonferroni-corrected *P*-values.

As expected, the Barry and Cass County sites were genetically differentiated from each other (*F_ST_* = 0.057), though this is likely an underestimate because we preferentially sequenced SNPs common in both populations (minor allele frequencies > 0.05). In a principal component analysis for both sites, the first axis of variation (which explained 14.3% of the variation in the data) divided the populations, with the potential migrant falling between them (*SI Appendix*, Fig. S2). Fine-scale population structure is revealed in principal component analyses conducted separately for each site (Fig. 2 *A* and *B*). In Barry County, PC2 (3.6% of variation explained) is associated with changes in latitude. In Cass County, PC2 (3.8% of variation explained) is associated with changes in longitude. At both sites, the direction associated with genetic clustering is roughly correlated with the distribution of riparian habitat.

To determine whether this spatial pattern was the result of kinship clustering, we compared pairwise geographic distances between related and unrelated individuals. Individuals recaptured in multiple locations were assigned a single location that was the centroid of all their capture locations. Parents and offspring, as well as pairs of parents, were more likely to be closer together compared to pairs of unrelated individuals with no offspring (Fig. 2 *C* and *D*, Bonferroni-corrected *P* for all tests < 0.005). This mirrors a pattern of isolation by distance (43, 44) seen in the genetic data, where more genetically similar individuals are, on average, found closer together in geographic space (*SI Appendix*, Fig. S3).

### Inbreeding Explains Reduced Individual Survival.

**Fig. 3.**
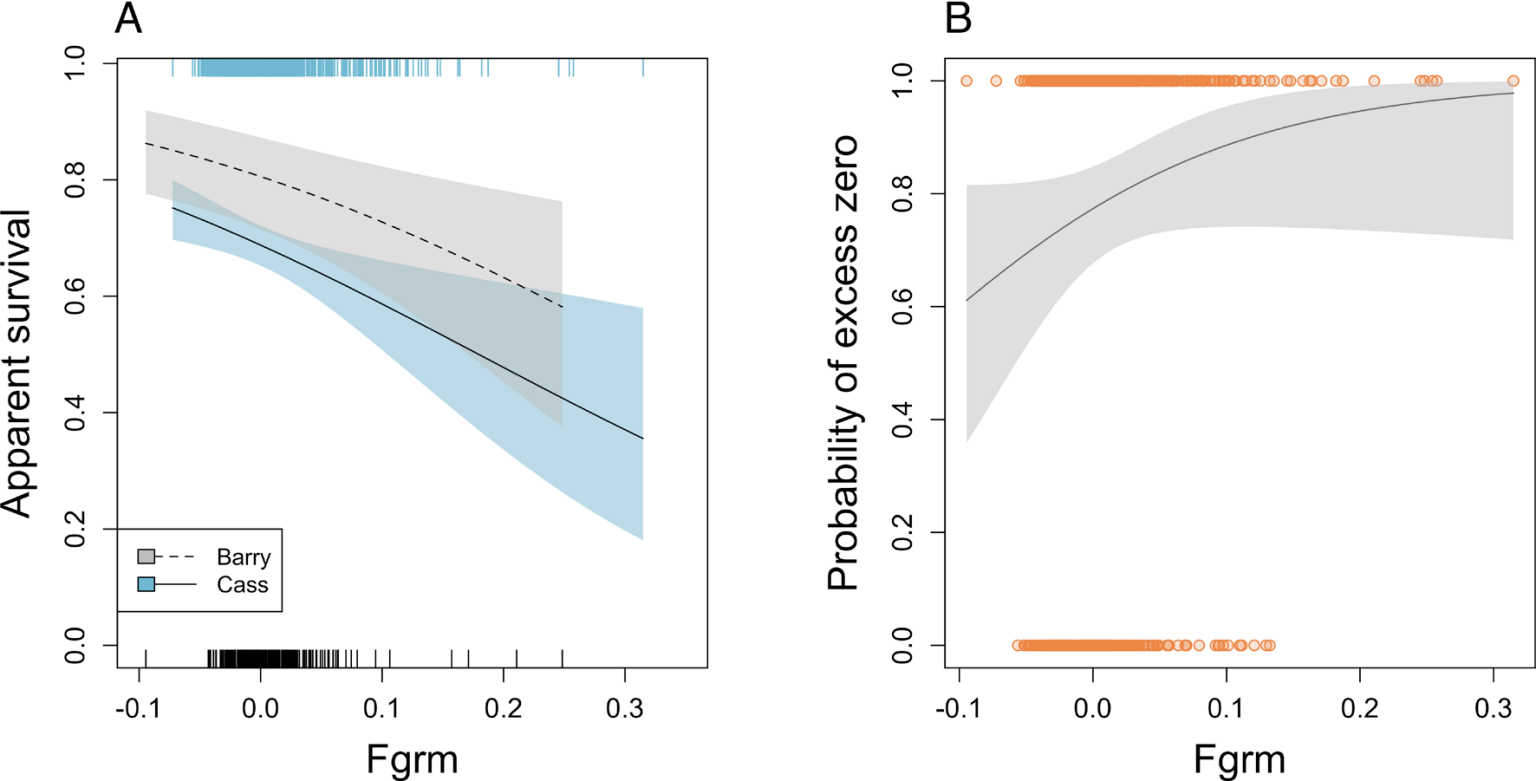
(*A*) Effect of inbreeding, as measured by *F_grm_*, on annual apparent survival from capture–recapture models of eastern massasaugas from Barry (dashed line) and Cass (solid line) counties. Shaded bands represent 95% CI. Internal ticks mark observed values of *F_grm_* for Barry (black) and Cass (blue) counties. To facilitate site comparisons, variation in snout–vent length (SVL) was controlled for by holding it at 52 cm (moderately sized adults at both sites). (*B*) Effect of *F_grm_* on the probability of excess zeros in number of pedigree offspring in the zero-inflated model. The solid line represents the predicted probability of offspring. The shaded band represents the 95% CI. Orange points show individuals that did (zeros) and did not (ones) have offspring.

We evaluated the effect of *F_grm_* on apparent survival by building a set of candidate Cormack–Jolly–Seber (CJS) models that included 1,591 captures (Cass: *n* =1,188; Barry *n* =403) of 1,001 unique individuals (Table 1, Cass: *n* =754; Barry *n* = 247) and determined the top-ranked model using AIC_c_. The top-ranked CJS model included negative additive effects of site and *F_grm_* and a positive additive effect of snout–vent length (SVL) to explain annual apparent survival (Table 1 and *SI Appendix*, Figs. S4 and S5). Barry County individuals had higher survival than Cass County individuals. To assess the effects of inbreeding on annual survival, we controlled for body size by setting SVL to 52 cm, which is the SVL of moderately sized adult males and females at both sites. Cass County adult annual survival decreased from 0.75 to 0.36 as *F_grm_* increased from −0.07 to 0.32 (Fig. 3). Barry County adult annual survival decreased from 0.86 to 0.58 as *F_grm_* increased from −0.09 to 0.25 (Fig. 3). The predicted apparent annual survival of the 5% most inbred individuals in each population decreased by 13.80% at Cass County and 9.46% at Barry County compared to the rest of the population.

**Table 1.** Survival models: Candidate set of Cormack–Jolly–Seber models constructed from capture–recapture data collected from eastern massasauga populations in Cass and Barry counties between 2009 and 2023

Survival Models	Δ AIC_c_	$\omega_{i}$	k	−2(log) L
ϕ (site + SVL + *F_grm_*) *p* (site * year + sex * year + AFpriorcap2)	0.00	0.315	46	3,173.86
ϕ (site + sex + SVL + *F_grm_*) *p* (site * year + sex * year + AFpriorcap2)	0.24	0.280	47	3,171.98
ϕ (site + sex + SVL * *F_grm_*) *p* (site * year + sex * year + AFpriorcap2)	0.53	0.242	48	3,170.14
ϕ (site * sex + SVL * *F_grm_*) *p* (site * year + sex * year + AFpriorcap2)	2.22	0.104	49	3,169.70
ϕ (sex + SVL + *F_grm_*) *p* (site * year + sex * year + AFpriorcap2)	5.28	0.023	46	3,179.14
ϕ (SVL + *F_grm_*) *p* (site * year + sex * year + AFpriorcap2)	5.53	0.020	45	3,181.52
ϕ (site + SVL) *p* (site * year + sex * year + AFpriorcap2)	6.96	0.010	45	3,182.95
ϕ (site + sex + SVL) *p* (site * year + sex * year + AFpriorcap2)	7.82	0.006	46	3,181.68
ϕ (SVL) *p* (site * year + sex * year + AFpriorcap2)	11.96	0.001	44	3,190.06
ϕ (sex + SVL) *p* (site * year + sex * year + AFpriorcap2)	12.31	0.001	45	3,188.30
Reproductive Output Models				
offspring ~ site + *F_grm_* + sex + PC2 + PC3 + PC4 + PC5 + PC6 + years	0.00	0.49	21	1,480.68
offspring ~ site + *F_grm_* * sex + PC2 + PC3 + PC4 + PC5 + PC6 + years	0.38	0.40	23	1,476.86
offspring ~ site + sex + PC2 + PC3 + PC4 + PC5 + PC6 + years	2.93	0.11	19	1,487.78

Each model contains apparent survival (ϕ) and recapture (*p*) submodels. Reproductive output models: Candidate set of zero-inflated negative binomial models. The same model was used for both conditional and zero-inflated components.

### Inbreeding Is Associated With Reduced Reproductive Output.

The inbreeding load (*-B*), estimated from a regression of the log of individual reproductive output against *F_grm_* with 826 genotyped individuals (Table 1, Cass: *n =* 589, Barry: *n =* 237), was 1.13. To further evaluate the effect of *F_grm_* on total reproductive output, we constructed zero-inflated negative binomial models. These models incorporate a conditional and zero-inflated component to account for an excess of true and false zeros in the number of pedigree offspring (45). We expect true zeros to occur because of reproductive skew and false zeros to represent individuals never captured during the study. The top-ranked model from AIC_c_ comparison included effects of site, principal components (PCs) 2 to 6 of the genetic PCA, years an individual could have contributed to the pedigree, sex, and *F_grm_* in both the conditional and zero-inflated components of the model (Table 1). Similar to accounting for population structure in genome-wide association studies, PCs account for patterns of shared ancestry within a site. The lowest-ranked model was the model that did not include *F_grm_* as a predictor.

Individuals with higher inbreeding were more likely to have zero pedigree offspring (i.e., excess zeros), whereas snakes that had more years with the potential to contribute to the pedigree (i.e., lived longer after reaching reproductive age) were less likely to have zero offspring (Fig. 3 and *SI Appendix*, Fig. S6). In the zero-inflated component of the model, an increase of 0.05 in *F_grm_* is associated with a 50.8% decrease in the odds of having any offspring in the pedigree. The predicted probability of having offspring of the 5% most inbred individuals in each population decreased by 13.23% compared to all other individuals. Years contributing to the pedigree, site (Cass), and PC6 had a positive effect on the total number of offspring produced (*SI Appendix*, Figs. S6 and S7). Inbreeding also had a negative effect on number of offspring, but the 85% CI overlapped zero, indicating it is not an important model parameter (46).

To address the possibility that spatial correlations in inbreeding and reproductive output might bias results, we compared *F_grm_* between nearby (within 25 m) individuals with and without offspring. On average, individuals without offspring have higher *F_grm_* than nearby parents (*t* = 2.70, df = 334, *P* = 0.007), but do not have significantly different *F_grm_* than other nearby individuals without offspring (*t* = −0.084, df = 409, *P* = 0.93). Furthermore, the difference in inbreeding between nearby pairs with and without offspring is greater than the difference between pairs of individuals without offspring (*t* = −2.03, df = 723.46, *P* = 0.043).

## Discussion

We documented patterns of inbreeding and inbreeding depression in two wild populations of the threatened eastern massasauga. While most individuals in our study were not inbred relative to the population as a whole, some individuals exhibited elevated levels of inbreeding, and inbreeding was associated with lower survival and reproductive success. We found evidence of fine-scale population structure within each site, which is a potential mechanism generating nonrandom mating and reduced individual fitness due to inbreeding. Previous studies on eastern massasaugas and other vipers have documented the accumulation of putatively deleterious variants that likely contribute to inbreeding depression (47) as well as physical abnormalities and decreased litter size thought to be a consequence of inbreeding (48–50). This study links inbreeding and fitness to provide direct evidence of inbreeding depression in rattlesnakes. Our results contribute to a growing body of work that suggests that, as predicted by experimental literature (12), inbreeding depression in wild animal populations is widespread and especially concerning given contemporary levels of habitat loss and fragmentation (3).

### Extent of Inbreeding.

While most individuals genotyped as a part of this study have inbreeding values near zero, indicating little to no inbreeding, more than 7% of individuals at both sites have *F_grm_* values above 0.05, a value known to cause detectable inbreeding depression in other taxa (51). Due to our large sample sizes, we were able to directly observe instances of close-kin mating in the pedigree (Fig. 1). For example, the most closely related parents were outbred full siblings. More individuals had elevated genomic inbreeding compared to pedigree inbreeding, likely due to the limited timescale captured by the pedigree (5).

### Fine-Scale Spatial Structure Generates Inbreeding Through Nonrandom Mating.

We found fine-scale spatial population structure within both sampled populations of eastern massasaugas (Fig. 2). Fine-scale spatial genetic structure and spatially nonrandom mating have been connected to inbreeding in simulations and marsupials (52) and a decrease in fitness in outcrossing plants (15). However, spatially random mating can act to prevent inbreeding even in the presence of spatial structure (53). Here, we show both spatial genetic structure and that close-kin pairs and parents are not spatially random, indicating a link between spatial structure and inbreeding.

Several facets of massasauga life history could generate spatial structure and inbreeding: Eastern massasaugas have limited dispersal, high fidelity to overwintering sites (54), and neonates return to hibernacula near where they are born to overwinter (55). Additionally, male snakes often return to specific areas each mating season to look for mates (56). Thus, even though we observed individual snakes having moved up to 960 m between capture locations in this study, spatially restricted mating and parturition could generate spatial genetic structure. Anthropogenic land use changes reduce dispersal between populations (30, 31, 57), perhaps increasing spatial structure within suitable habitat. Understanding the demographic and dispersal histories of these populations could help elucidate the role anthropogenic barriers have played in the development of spatial structure within populations. These results highlight that not all small populations are at equal risk of inbreeding depression; some species, like eastern massasaugas, could be especially susceptible because of their life history traits.

### Fitness Consequences of Inbreeding.

We detected a significant negative relationship between inbreeding and both survival and reproductive success in eastern massasaugas. Inbreeding had a strong negative effect on survival for both sexes at both sites. The precise mechanism of this relationship is unknown; inbred snakes could suffer higher mortality from predation, could be more susceptible to diseases, like Ophidiomycosis (snake fungal disease), which is known to be present, but rare, at these sites (58), could experience greater overwintering mortality compared to noninbred individuals and/or could experience other intrinsic physiological factors leading to decreased survival. It is also possible that viability selection against inbred neonates could filter inbred individuals out of the population before they are detected, causing us to underestimate the extent of inbreeding depression on survival (59).

We found that inbred parents do not have significantly fewer offspring compared to noninbred parents, but inbred individuals are less likely to have any offspring represented in the pedigree at all. We expect individuals to have no pedigree offspring due to both reproductive skew (“true zeros”, i.e., lower reproductive success) and because of uncaptured individuals (“false zeros”, i.e., offspring die before capture or have lower capture probabilities). We account for differences in survival between individuals by using the number of years an individual could have contributed to the pedigree as a covariate; thus, the relationship between inbreeding and reproductive success is not driven by differences in parent survival. However, most individuals are first captured as adults, making it impossible to disentangle reproductive success and neonate mortality. Inbred snakes may have fewer mating opportunities, smaller litter size, higher rates of failed gestation, reproduce less frequently, and/or have offspring with higher rates of neonate mortality.

While the genetic architecture underlying inbreeding depression in these populations is unknown, the presence of inbreeding depression suggests the expression of deleterious recessive alleles. Ochoa and Gibbs (2021) found that eastern massasaugas in small populations at their range edge have fewer putatively deleterious variants than their less inbred congener *S. tergeminus*, suggesting that small historical population sizes have led to purging of some deleterious recessive alleles in eastern massasaugas (26). It is interesting to note that our focal populations are in the core of the extant range and larger than most range-edge populations, leading to the possibility that they harbor comparatively greater deleterious variation. A relationship between inbreeding and fitness could also be generated via heterosis, whereby outbred individuals with high survival and reproduction drive the relationship between inbreeding and fitness. However, we did not observe many individuals with negative *F_grm_*, indicating that heterosis is unlikely to produce the observed association.

In this study, we primarily quantify the extent and impact of inbreeding due to nonrandom mating on eastern massasauga fitness. We expect that given small effective and census population sizes and likely recent isolation, these populations have also lost genetic diversity due to drift, which could further reduce the fitness of all individuals in the population as well as limit adaptive potential (47). This would be an interesting avenue for future investigation.

### Conclusions and Conservation Impacts.

Understanding how fragmented and small populations persist on the landscape and identifying future threats to their persistence is essential. We document evidence of inbreeding depression in eastern massasaugas and in rattlesnakes in general. Long-term population monitoring is essential for understanding threats to, and subsequent conservation of, imperiled populations. While previous research has found that these populations are stable (60, 61), our results indicate that there is sufficient deleterious variation in the gene pool to negatively impact fitness and that this phenomenon might be common in eastern massasaugas in structured and isolated populations due to their life history. This highlights the importance of maintaining or restoring connectivity between local populations and investigating the implementation of genetic rescue for long-term population health.

## Materials and Methods

### Sample Collection.

This study focuses on two populations of eastern massasauga rattlesnakes in southwestern MI, 86 km apart: Cass and Barry Counties. Detailed descriptions of these populations and our sampling and processing methods are previously published (28, 61) and in the supplemental methods. All data and code are available in a Dryad repository (62).

### RAPTURE Genotyping.

We extracted DNA from all unique blood samples collected over 11 years at Cass County (2009–2021, no 2020) and 10 years at Barry County (2011–2023). Twelve BestRAD libraries were prepared using custom RAPTURE baits and sequenced, containing 1,056 individuals (30 individuals sequenced as part of multiple libraries to act as technical duplicates) (35, 63). A full description of our RAPTURE baits and bioinformatic pipeline is in the supplemental methods. Briefly, we aligned sequencing data to the eastern massasauga reference genome (47) using BWA mem v. 07.17 (64). Alignments were filtered and sorted using Samtools v. 1.9 (65). We used the Stacks ref pipeline to call SNPs and remove PCR duplicates. We filtered SNPs using bcftools v.1.9.64 (66) to retain SNPs with more than seven reads in 90% of individuals and with genotype quality scores greater than 19 in 90% of individuals. We filtered out SNPs with excess heterozygosity (*P* < 0.05) and filtered to retain one randomly selected biallelic SNP per targeted genomic region at least 100 kbp apart on the genome. Using custom R code, we removed SNPs with more than 10% missing data and removed individuals with greater than 20% missing genotype calls or that had uneven distributions of reference and alternate allele read counts indicative of sample contamination. For principal component analysis, we filtered out SNPs with a minor allele frequency below 0.05. We filtered out SNPs with a minor allele frequency less than 0.1 separately for each site to reconstruct pedigrees. We calculated N_e_ for each population using the strataG v. 2.5.01 package in R with individuals from the same estimated birth year (Cass: 2013, Barry: 2015, see Supplemental Methods), using linkage disequilibrium measured by r^2^ and calculated 95% CI using jackknife resampling (36, 37). Because these populations violate assumptions of random mating, our estimates may be biased downward (67). We used the R package hierfstat v. 0.5-11 to calculate Weir and Cockerham’s estimate of *F_ST_* (68, 69).

### Spatial Population Structure.

For individuals with multiple captures, we calculated the centroid of their capture locations to use in spatial analyses using the “st_centroid” function from the sf package v. 1.0-9 in R (70). If an individual was only captured once, that capture location was used in spatial analyses. To visualize genetic structure within and between sites, we used principal component analyses implemented in custom R scripts. To assess the relationship between genetic and geographic distance, we calculated pairwise π at polymorphic sites, a measure of genetic differences between pairs of individuals, using custom code and plotted it against pairwise geographic distance. We compared distance distributions between closely related individuals (dam–offspring, sire–offspring) and parents (pairs with offspring in the pedigree) with the distribution of distances between unrelated individuals without offspring using Kolmogorov–Smirnov tests. All pairwise geographic distances were calculated using the “st_distance” function from the package sf v.1.0-9 in R (70).

### Quantifying Inbreeding and Genetic Diversity.

To estimate individual inbreeding, we calculated *F_grm_* using custom code in R (41, ${\hat{F}}_{i}^{III}$ in 42). For a given individual, *F_grm_* is defined as

$$F_{\mathrm{grm}}=\frac{1}{L}\sum_{i}^{L}\frac{x_{i}^{2}-\left(1+2p_{i}\right)x_{i}+2p_{i}^{2}}{2p_{i}(1-p_{i})},$$

where $p_{i}$ is the population allele frequency at locus i and $x_{i}$ is the number of copies of the reference allele in the focal individual. Multilocus heterozygosity and *F_grm_* are highly correlated (23). To measure inbreeding directly from the pedigree (see below), we calculated pairwise kinship coefficients using the “CalcRped” function in Sequoia v. 2.9.0 in R and identified related individuals with shared offspring in the pedigree (38). We calculated pedigree-based inbreeding coefficients using the “calcInbreeding” function in the pedigree v. 1.4.2 R package (71).

### Assessing Inbreeding Depression: Survival.

We evaluated the effects of site, sex, SVL, and *F_grm_* on apparent survival using Cormack–Jolly–Seber (CJS) models implemented in the Program MARK v. 10.1 (72). We accounted for missing surveys in Barry (2009 and 2010) and Cass counties (2020) by creating dummy occasions and inserting “dots” (.) indicating the missing years for affected individuals’ capture histories. We evaluated CJS assumptions using U-CARE (73), replacing the dots with zeros, and stratified our capture histories into four site- and sex-specific groups. We detected significant transience and trap dependence for Cass County females, indicating an excess of snakes never detected after their initial capture and a trap-shy effect in recapture probabilities. We assumed the transience and trap dependence effects in Cass County females were caused by unmodeled heterogeneity in apparent survival and recapture probabilities, which we accounted for in the CJS models described below.

We treated sex and site as factors to explain apparent survival and recapture probabilities and *F_grm_* as a static continuous individual covariate to explain apparent survival. We accounted for the approximately biennial change in adult female recapture probabilities (61) using a function that assigned individuals a “1” if they were captured as an adult (i.e., ≥45 cm SVL) two years prior to the current year and a “0” if they were not captured two years ago as an adult (AFpriorcap2). To incorporate SVL as an individual-based temporally varying covariate in our CJS models, we constructed ten candidate models using the Fabens (74) formulation of the von Bertalanffy (75) growth model (*SI Appendix*, Table S3) (74, 75). We used individual records of date and length at initial and last capture, separated by ≥ 1 winter, to estimate the maximum length $\left(L_{\infty }\right)$ and growth coefficient (K) using nonlinear least squares in R version 4.3.2. We used AIC_c_ (76) adjusted for small sample size via the R package AICcmodavg (77) for model selection. Models including uninformative parameters or that were simply nested versions of more parameterized ranking models were considered noncompeting and discarded prior to assessing model support (46, 78). We used the top-ranked Fabens model to predict SVL for all individuals with missing SVL values (79).

We standardized and centered each covariate by subtracting its mean and dividing it by two SD so that continuous and binary regression coefficients were approximately on the same scale (80). We constructed ten candidate CJS models using additive and interactive effects of the factors and covariates (Table 1). For the global CJS model, the apparent survival submodel included interactions between site and sex and between SVL and *F_grm_* to explain apparent survival. The recapture submodel was highly embellished (42 parameters) to account for site differences in recapture and sampling effort and was included in all candidate models. It included interactions between site and year and between sex and year, and an additive adult female behavioral response to recapture (i.e., the AFpriorcap2 function). All candidate models were nested within the global model. We used the previously described model selection criteria to determine the most supported model.

### Pedigree Reconstruction.

Pedigrees for each site were reconstructed using the R package Sequoia, which assembles pedigrees based on genotype data, and approximate age and sex information. For individuals with unknown birth years, we approximated birth year using SVL and von Bertalanffy growth models (see Supplemental Methods, *SI Appendix*, Figs. S8 and S9). We used the inferred birth year +/- two years as minimum and maximum birth years. We calculated genotyping error rate by comparing genotypes between technical replicates and estimated CI using jackknife resampling, after which duplicates were removed from the dataset. We evaluated our pedigree construction by validating that known dam–offspring relationships at the Cass County site (*n* = 265) were called correctly. We also estimated our confidence in the reconstruction using the “EstConf” function in Sequoia (61).

We used the pedigrees to estimate individual reproductive success (total number of offspring) and corrected these numbers to account for offspring of gravid females that gave birth in captivity as part of another study (40) such that we only counted a captive-born offspring toward a mother’s reproductive success if that individual was recaptured after parturition or had offspring/grand-offspring of its own in the pedigree, indicating that it survived to adulthood. Of the offspring born in the lab, 46% were only recorded at birth and were not assigned offspring/grand-offspring in the pedigree. To determine whether there were edge effects in offspring count generated because we captured individuals within a defined spatial extent, we tested whether distance from the center of each site, measured as the centroid of all individual centroid locations, is a predictor of offspring number using linear regressions.

### Testing for Inbreeding Depression: Reproductive Success.

We measured inbreeding load (-*B*) by performing a linear regression between *F_grm_* and log offspring. To avoid negative infinity values, we added one to offspring count before taking the natural log. We included site and the number of years an individual could have contributed offspring to the pedigree as fixed effects. To test for a relationship between reproductive success and *F_grm_*, we built zero-inflated linear mixed models with a negative binomial distribution using the glmmTMB v. 1.1.10 package in R (81). We constructed three models to test the effect of *F_grm_* on the number of pedigree offspring. Our full model included site, sex, *F_grm_*, the number of years an individual could have contributed offspring to the pedigree, an interaction term between sex and *F_grm_*, and five principal components (PC2-PC6, PC1 captured the effect of site) on number of offspring (Table 1). The number of years an individual could have contributed to the pedigree accounts for varying reproductive opportunities between individuals due to variation in longevity and birth year (see Supplemental Methods). PC2 through PC6 were included to account for genetic relatedness between individuals, as variation in fitness likely exists between family groups (19). The interaction term between sex and *F_grm_* tests the hypothesis that inbreeding depression is more severe for the homogametic sex. We scaled all numeric predictors by dividing each predictor by its SD.

We included a zero-inflation term in our model to account for an excess of true and false zeros in the number of pedigree offspring (45). True zeros occur because of reproductive skew, and false zero represent individuals never captured. We used the same model for the conditional and zero-inflated components as our a priori expectation is that the same set of factors could contribute to both reproductive success and offspring count. We determined the most supported model using AIC_c_ comparison. To address potential spatial correlations confounding modeling results, we compared the distribution of differences in *F_grm_* between pairs of individuals without offspring and nearby (within 25 m) parents to the distribution of differences in *F_grm_* between pairs of nearby individuals both without offspring using a two-sample t test. We also compared the average difference in *F_grm_* between nearby pairs of parents and pairs of individuals both without offspring to zero using a one-sample *t* test.

## Supplementary Material

Appendix 01 (PDF)

## Data Availability

Processed capture-recapture field data and derived genetic sequence data as well as all scripts required to reproduce analyses in the paper are available via a Dryad repository (62). Raw sequencing data are available in the NCBI Sequence Read Archive (BioProject PRJNA1295676) (63). The eastern massasauga is a species of conservation concern throughout its range. Following best practices for listed species, we have restricted sensitive spatial and recapture information. Because many of our analyses rely on spatial information, Cartesian coordinates have been randomly shifted such that the geographic distances between individuals remains the same, but coordinates no longer represent the actual spatial location of the focal populations.
