# Supplementary material for: Inbreeding reduces fitness in spatially structured populations of a threatened rattlesnake
Source: Proc Natl Acad Sci U S A. 2025 Aug 18;122(34):e2501745122. doi: 10.1073/pnas.2501745122 (PMC12403008; doi:10.1073/pnas.2501745122)
Supplement: Supplementary file 1 — Appendix 01 (PDF) [file pnas.2501745122.sapp.pdf]

PNAS

Supporting Information for

Inbreeding reduces fitness in spatially structured populations of a threatened rattlesnake

Meaghan I. Clark, Eric T. Hileman, Jennifer A. Moore, Lisa J. Faust, Randall E. Junge, Brendan N. Reid, Danielle R. Bradke, Gideon S. Bradburd, Sarah W. Fitzpatrick

Corresponding author: Meaghan I. Clark
Email: meaghaniclark@gmail.com

This PDF file includes:

Supporting text
Figures S1 to S10
Tables S1 to S3
SI References

Supporting Information Text
Supplemental methods

Capture-recapture surveys. We conducted field surveys during the active season from 2009 through 2023. Data was collected from Cass County for 14 years (2009-2023, no 2020) and from Barry County for 13 years (2011-2023). Both sites are managed for eastern massasaugas (e.g., controlled burns, invasive species removal) and surrounding by a large proportion of agricultural or otherwise developed land cover. Sexual maturity for males and females is reached at approximately age three (1). The oldest individual of known age in the study lived to at least 12 years old. After recording location information, captured snakes were brought back to the lab where we recorded sex (2) , snout-vent length (SVL (cm), (3), and other relevant morphometrics. Snakes were individually marked with passive integrated transponder (PIT) tags. We collected blood from the caudal vein for genetic analyses and released individuals at their site of capture. 30 gravid females at the Cass County site were held in the lab until partition as part of a separate study (4, 5). We included these individuals and their offspring to verify our pedigree reconstruction.

Initial library preparation. To identify polymorphic loci in our focal populations, 64 individuals were included in an initial RAD library (39 from Barry County; 25 from Cass County) (Ali et al 2016). We submitted a single library for sequencing with paired-end 150 bp reads on an Illumina HiSeq 4000 at Michigan State University's genomic core facility. Sequencing yielded 310 million sequence reads and quality checks revealed high Q-scores (85% > Q30), indicating this library was successful.

Initial library bioinformatics. Raw sequence reads from the initial RADseq library were processed through two separate bioinformatic pipelines: Stacks (6) and ipyrad (7). Reads were filtered for quality and clustered both within and among individuals in order to identify sequences containing single nucleotide polymorphisms (SNPs). For the ipyrad analysis, reads were mapped to a draft genome for the eastern massasauga rattlesnake (8). Mapping reads to a reference genome improves genotyping accuracy and facilitates a wider set of analyses that take advantage of precise genomic location information. Average read depth across all samples for sequences mapped to the reference genome was 15X and in total 11,862 individual loci containing SNPs were identified.

To identify target SNPs for RAD-based capture assays, 64 individuals were included in an initial BestRAD library (39 from Barry County and 25 from Cass County). We sequenced the library on an Illumina HiSeq 4000 with 150 bp paired-end reads at the Michigan State University Core Genomics Facility. This initial library was used to identify 3,391 putative neutral candidate SNPs with minor allele frequencies > 0.05 in both populations, spaced at least 100 kbp apart on the genome. Custom baits to target these SNPs were designed by Arbor Biosciences (Ann Arbor, MI).

Bait design. An initial candidate set of loci was generated from the 64-individual pilot data set that included SNPs proximate to venom-related genes (*svmp*, *svsp*, *pla2*, and *myotoxin*) and immune-related genes (*MHCI*, *MHCII*, and *defensin*) as well as loci with no association with known functional genes. From the SNPs located within loci with no known functional role, a subset of candidate SNPs (3,391 putatively neutrally-evolving SNPs) with known positions on the draft reference genome were used to design baits for sequence capture, retaining baits with fewer highly repetitive sequences and more unique BLAST hits based on criteria previously used by Arbor Biosciences. Baits were designed in sets of three eighty-base oligonucleotides tiled along an approximately 120-base RAD locus to maximize capture probability and efficiency.

RAPTURE genotyping. We extracted DNA from all unique blood samples up until 2019 at Cass County and 2021 at Barry County using Qiagen DNeasy Blood and Tissue kits. DNA was quantified using a Qubit 2.0 and quality was assessed using agarose gels. Twelve BestRAD libraries were prepared containing 1,056 individuals (30 individuals sequenced as part of multiple libraries to act as technical duplicates) (9). Libraries were pooled for bait capture, resulting in

three final pooled libraries. To validate even representation of individual libraries within pools, we first sequenced the pooled libraries on one lane of a MiSeq v2 nano. We ran libraries on a single lane of a NovaSeq 6000 S4 flow cell at the Michigan State University Core Genomics Facility.

We demultiplexed sequence data using STACKS v. 2.59 (6) and aligned them to the eastern massasauga reference genome (8) using BWA v. 07.17 (10). We used Samtools v. 1.9 (11) to index and sort alignments, as well as filter out unpaired reads. We used the Stacks ref pipeline to call SNPs and remove PCR duplicates using the following parameters: --rm-pcr-duplicates -X "populations: -p 2 -r 0.75". We filtered SNPs using bcftools v.1.9.64 (12) to retain SNPs with more than seven reads in 90% of individuals and with genotype quality scores greater than 19 in 90% of individuals. We filtered out SNPs with excess heterozygosity ($p < 0.05$) using the "HWExactStats" function in the HardyWeinberg R package v. 1.7.5 (13). We also filtered to retain one randomly selected bi-allelic SNP per targeted genomic region. Finally, using custom R code, we removed SNPs with more than 10% missing data, and removed individuals with greater than 20% missing genotype calls or that had uneven distributions of reference and alternate allele read counts indicative of sample contamination (14). For principal component analysis, we filtered out SNPs with a minor allele frequency below 0.05. For pedigree reconstruction, we filtered out SNPs with a minor allele frequency less than 0.1 separately for each site. We calculated effective population size (N_e) for each population using the strataG package in R, using linkage disequilibrium based on Pearson correlation approximation as in Waples et al. (2016) (15, 16). We estimated 95% confidence intervals around N_e estimates using jackknife resampling.

Inference of individual birth years. To estimate individual birth years, we predicted individual SVL with the von Bertalanffy (Fabens) top model (Supplementary Table 3). Specifically, we predicted SVL backward in time for individuals first captured with an SVL < asymptotic size, assuming a minimum size of 12 cm, the size of the smallest neonate observed at either site. We refined the backward predictions of SVL (also needed to estimate longevity; see below) based on our best estimates and knowledge of when individuals were age zero. To do this, we used the following rules: (1) If an individual was captured at age zero, we used the recorded (measured) SVL at that capture event. (2) If an individual was not captured at age zero, we evaluated their predicted SVL in the year prior to the year that their first predicted or recorded SVL was > 24.5 cm (Cass) or > 21.8 cm (Barry). These sizes represent the maximum SVLs of known age zero captures; thus, we assumed that an SVL > 24.5 cm (Cass) or > 21.8 cm (Barry) indicated an individual was age 1 or older. If the predicted SVL in the year prior was less than the mean SVL at age zero (19.2 cm for Cass and 19.3 cm for Barry), then we increased the individual's age zero SVL to the mean. If an individual was not captured at age zero, but their predicted SVL at age zero was > the mean size at age zero, then we kept the predicted age zero size. We also checked for individuals that were known to be age one at their first capture and were smaller than the maximum SVL of known age zero captures. We assigned these individuals the mean size at age zero at the occasion prior to their first capture. This correction ensured that individuals known to be age one were not incorrectly treated as age zero at age one. We used the predicted year an individual was age zero as their estimated birth year.

Calculation of longevity and years contributing offspring to the pedigree. To account for differences in survival and age at the end of the study when assessing the impact of F_{grm} on reproductive output, we calculated the number of years an individual could have contributed offspring to the pedigree. We used the top ranked CJS model to predict yearly cumulative survival probabilities based on SVL, site, and F_{grm} for every year starting in an individual's estimated birth year. We assumed an individual had died or left the population when its cumulative survival probability was less than 0.005. For individuals who were considered deceased before the last year new captures were included in pedigree reconstruction (2019 for ELF, and 2021 for PCCI), we calculated individual longevity (inferred death year - inferred birth year). For individuals who were considered alive during the last year individuals were included in pedigree reconstruction, we used that year as an upper cut off for when that individual could have contributed to the pedigree ("last year contributing", [2019 or 2021] – inferred death year). We do not account for any variation in number of years it took an individual to become sexually mature.

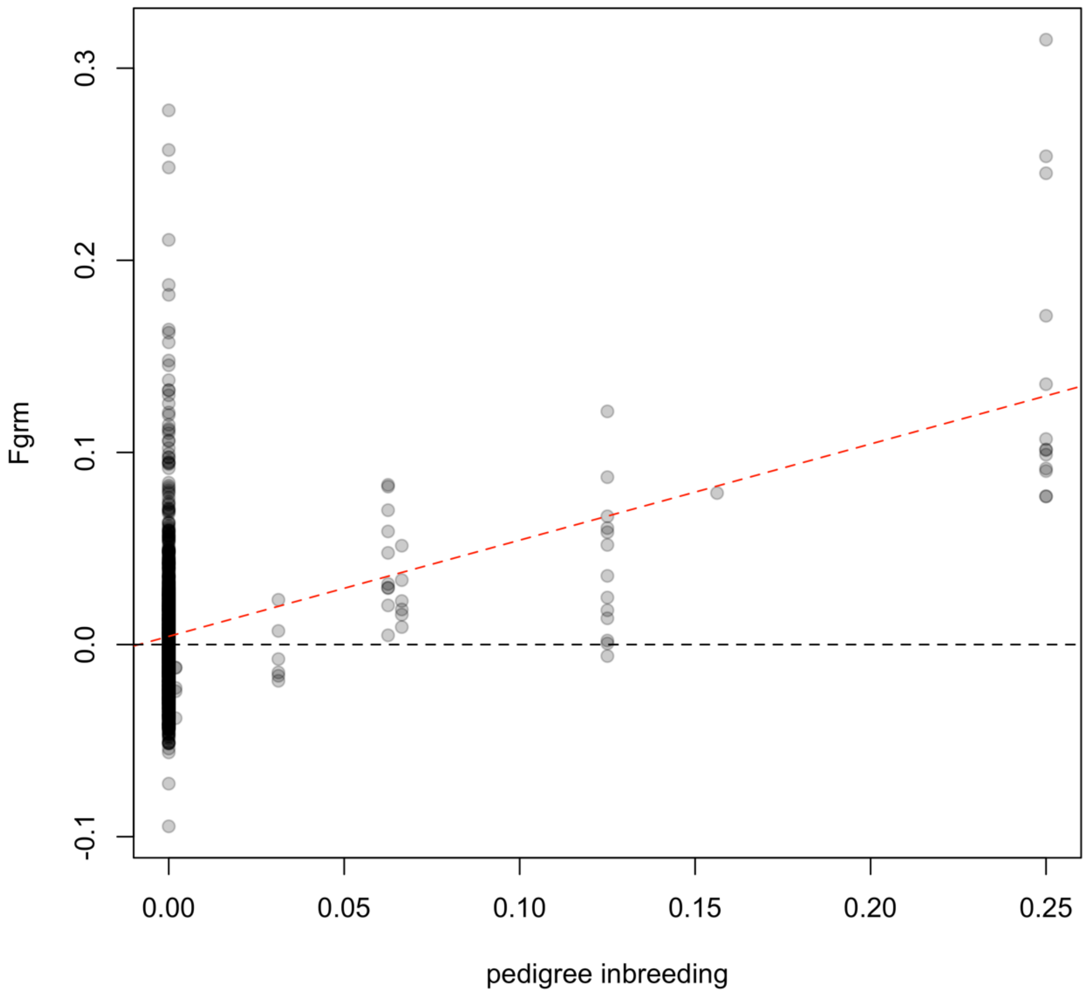

Fig. S1. Plot shows the relationship between pedigree inbreeding and F_{grm} for eastern massasaugas at two sites in Michigan **(r(1034) = 0.394, $p < 0.001$)**.

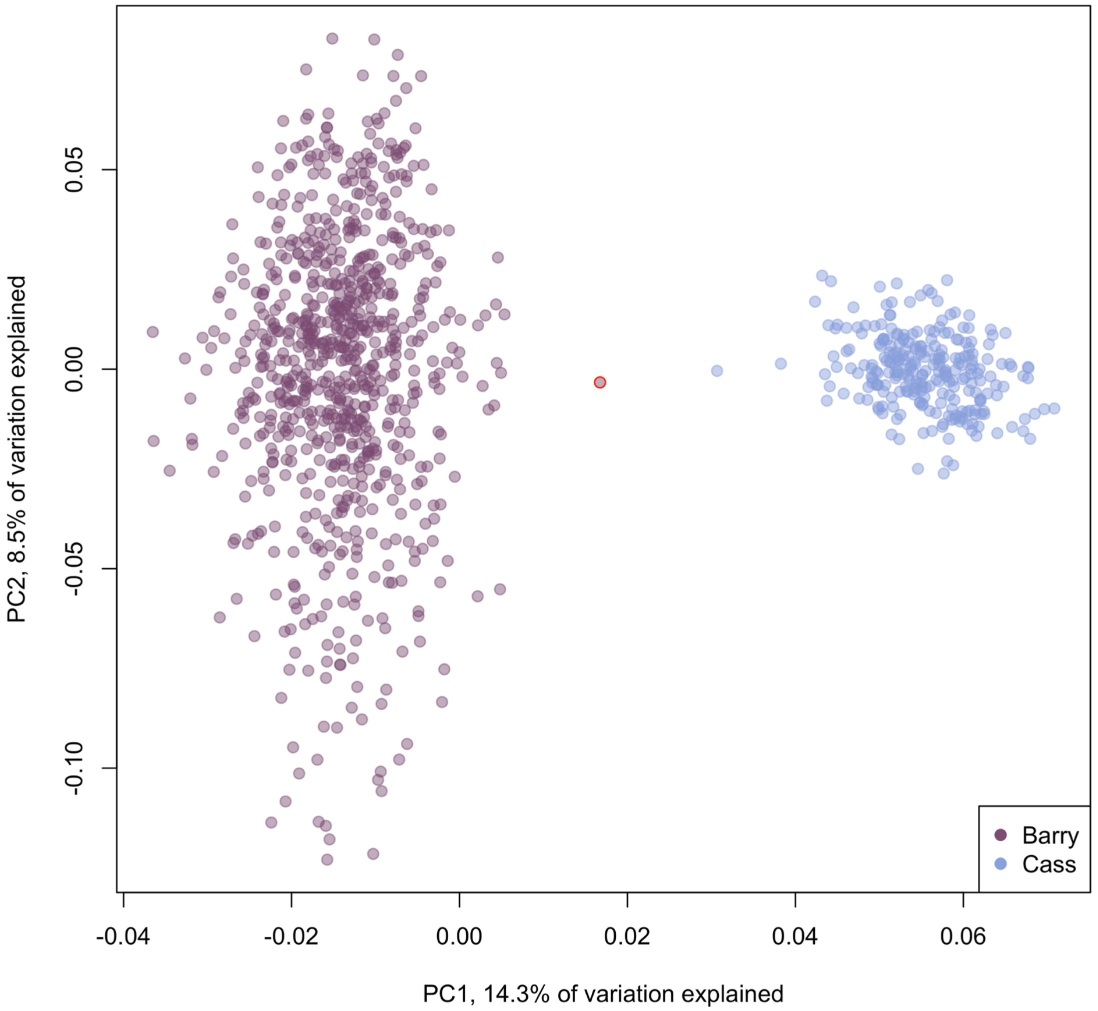

Fig. S2. Principal component analysis of eastern massasaugas at two sites in Michigan. Individual circled in red is the potential migrant in Cass County that was removed from downstream analyses.

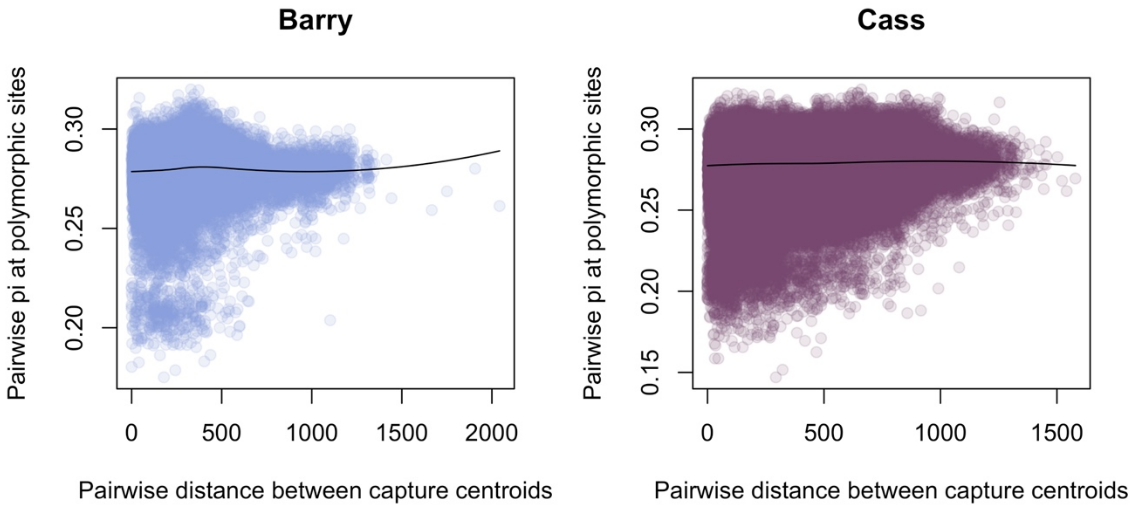

Fig. S3. Plots show the relationship between pi at polymorphic sites and pairwise distance between centroid locations in meters for eastern massasaugas at two sites in Michigan, USA. Line represents smoothed loess line.

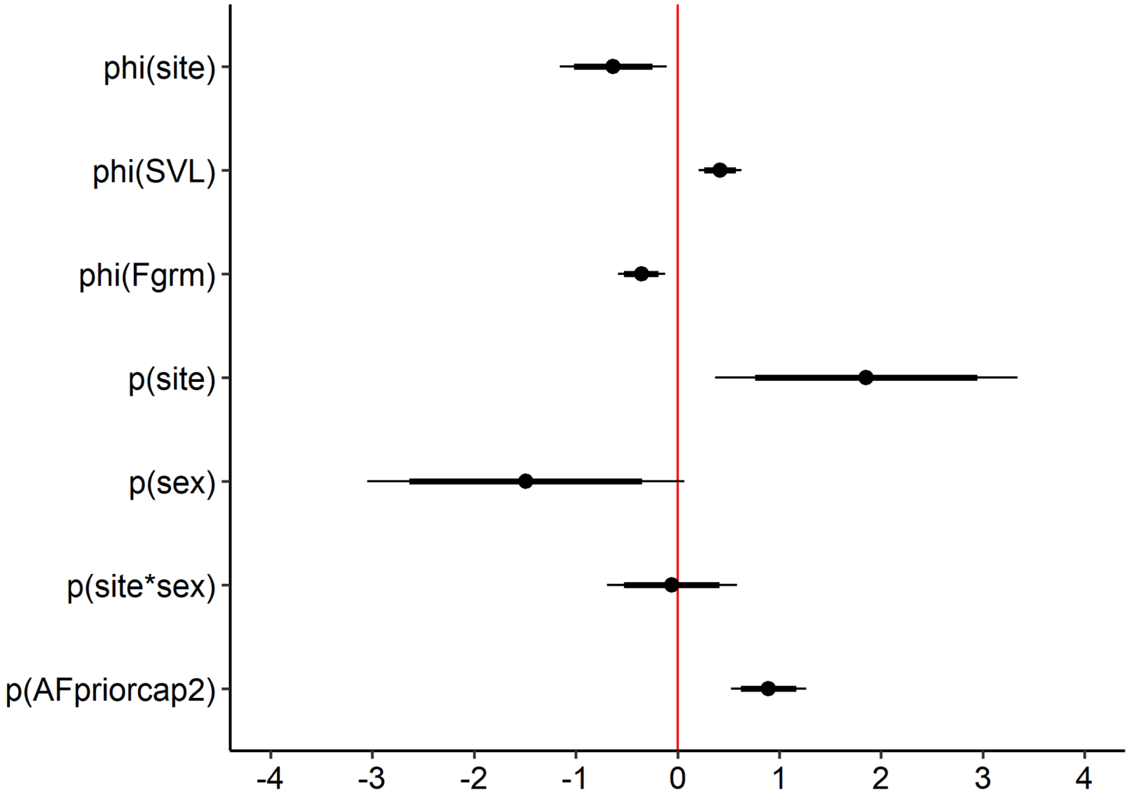

Figure S4. Standardized and zero-centered (red line) mean effect sizes (circles) of beta coefficients and their associated 85% (thick bars) and 95% (thin bars) confidence intervals based on the top-ranked Cormack-Jolly-Seber model. The model was fitted to capture-recapture data collected in Cass and Barry counties, MI, from 2009–2023. Shown are the effects of site (Cass), SVL, and F_{grm} on annual apparent survival and the effects of site (Cass), sex (male), and a (biennial) behavioral response to recapture for adult females (AFpriorcap2). Site (Barry) and sex (female), not shown, are the intercepts for survival and recapture probabilities, respectively.

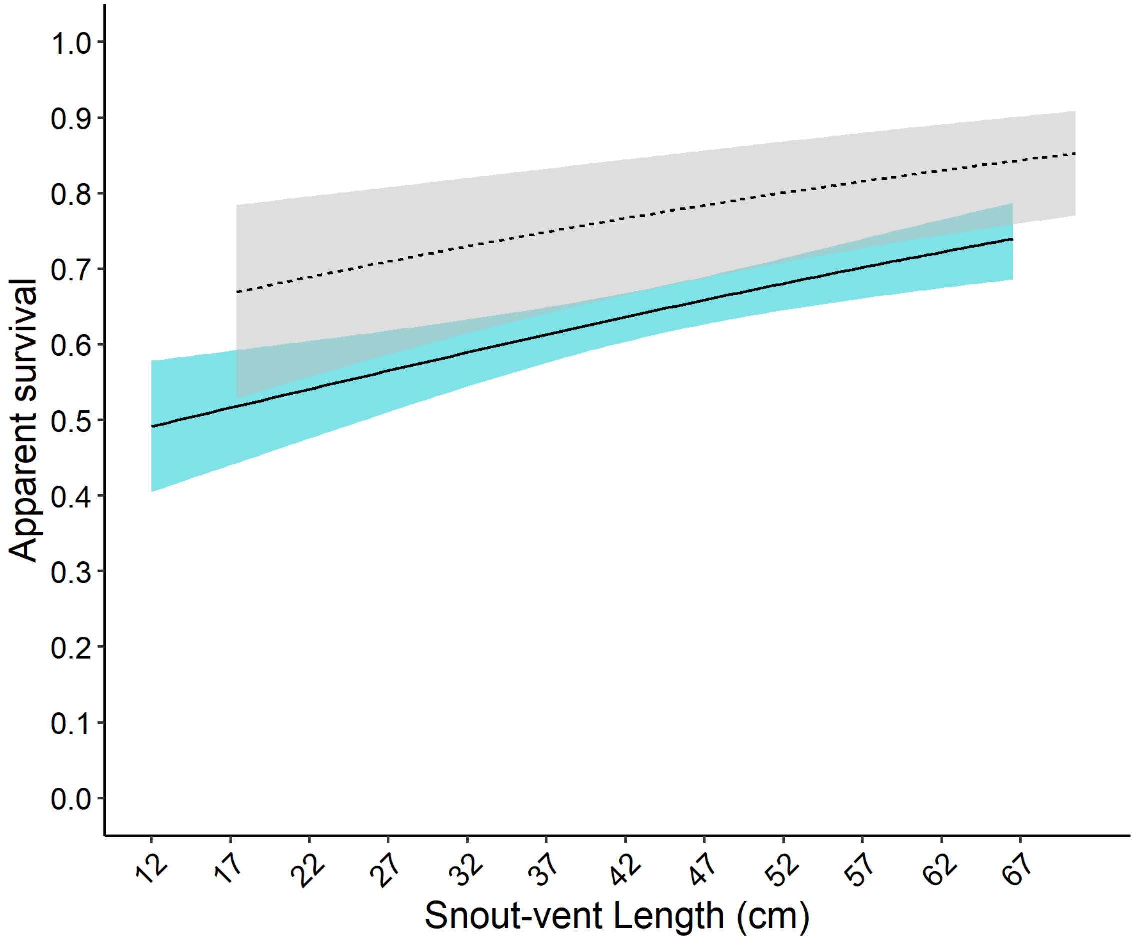

Figure S5. Effects of snout-vent length on annual apparent survival for eastern massasaugas from Barry (dashed line) and Cass (solid line) counties. Shaded bands represent 95% confidence intervals. To facilitate site comparisons, variation in F_{grm} was controlled for by holding it at its mean 0.00728.

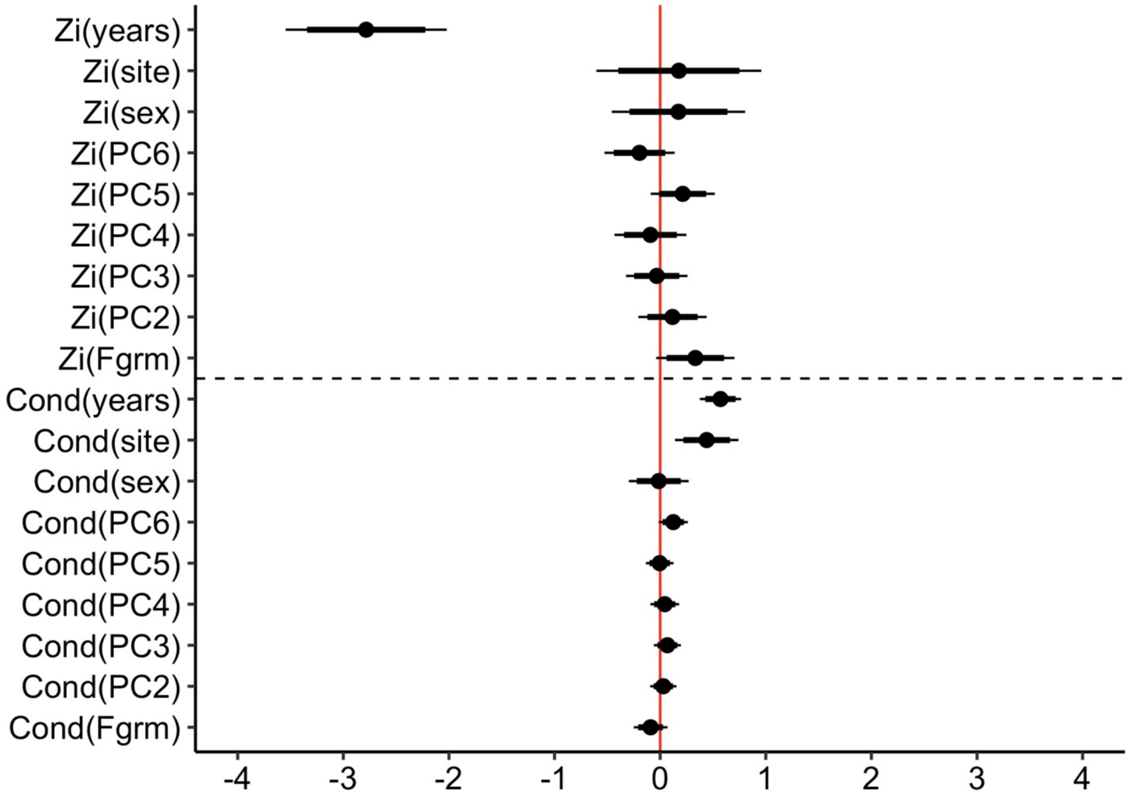

Figure S6. Standardized effect sizes (circles) and their associated 85% (thick bars) and 95% (thin bars) confidence intervals based on the top-ranked zero-inflated negative binomial model. Effect sizes above the dashed line are for the zero-inflated component of the model, and effect sizes below the dashed line are for the conditional component of the model. Shown are the effects of site (Barry), sex (males), years an individual contributed to the pedigree, genetic principal components 2-6, and F_{grm} on number of pedigree offspring.

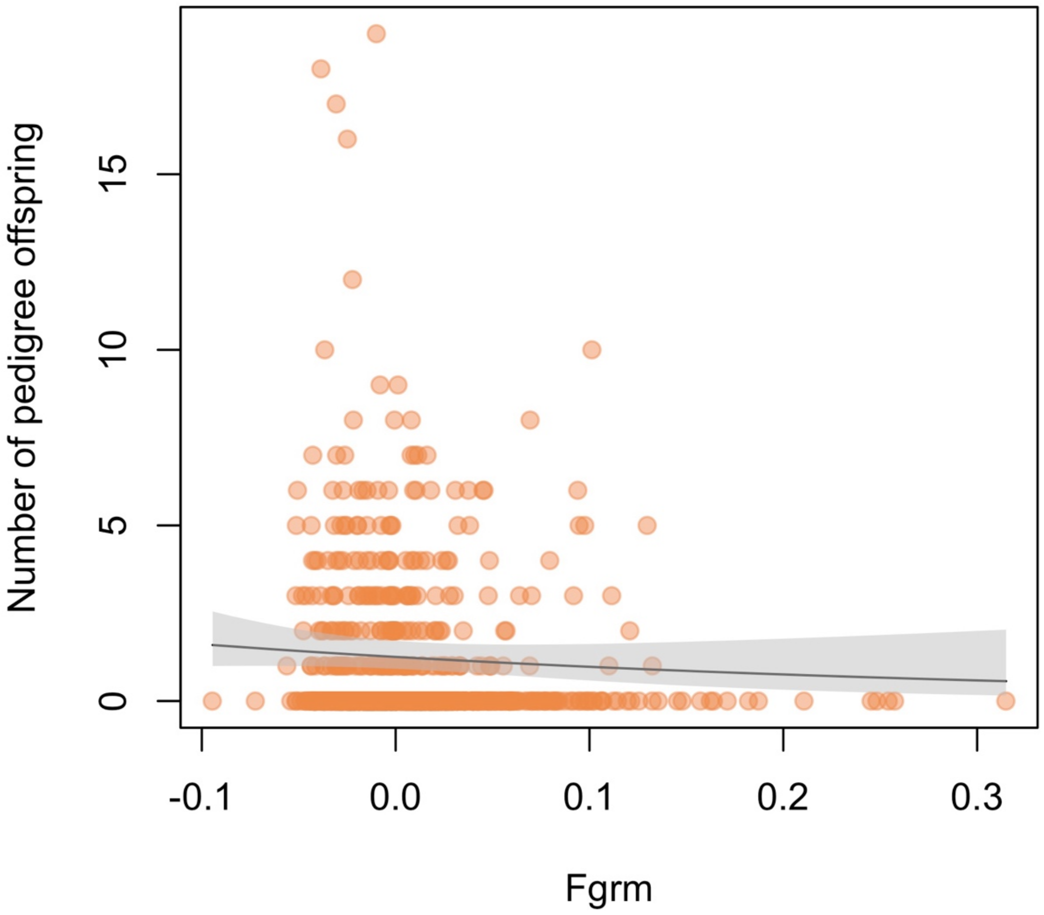

Figure S7. Effect of F_{grm} on number of offspring assigned in the pedigree. The solid line represents the predicted number of offspring. The shaded band represents the 95% confidence interval. Orange points are the data used to fit the model.

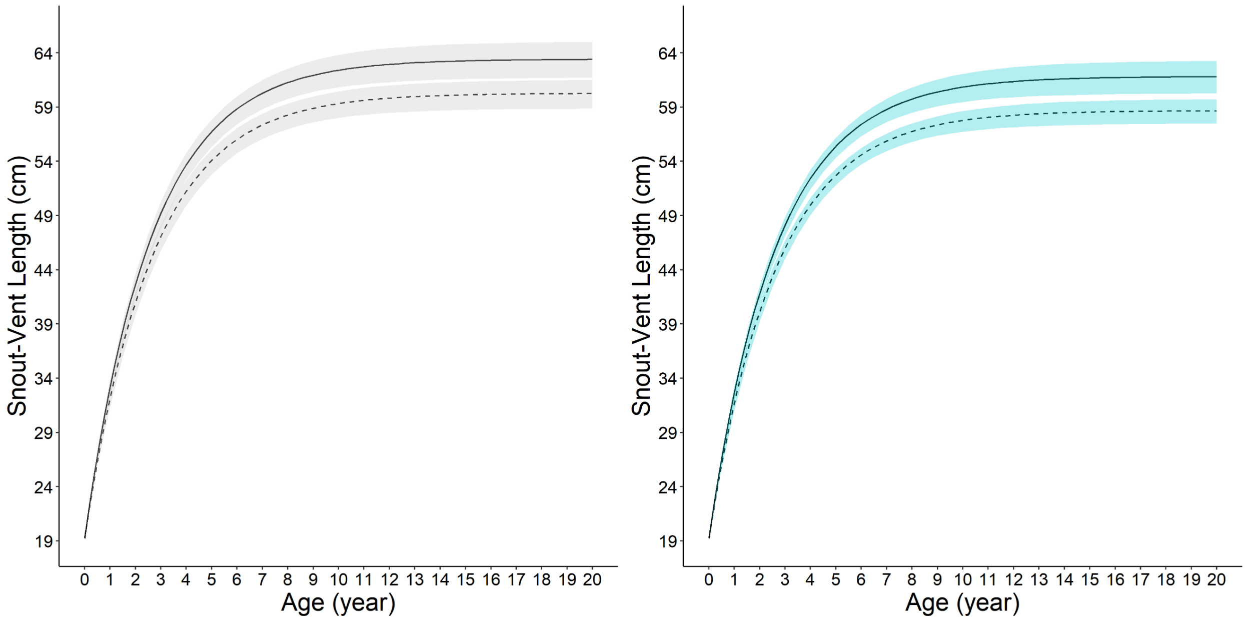

Figure S8. Eastern massasauga growth and asymptotic size for males (solid line) and females (dashed). Shaded bands represent 95% confidence intervals for Barry (left) and Cass (right) counties. Growth was constant between sexes and populations (*K* =0.378, 95% CI = 0.349–0.408), whereas asymptotic size was larger for males and females in Barry County. Size at birth (age 0 = 19.24 cm) was calculated from the average neonate snout-vent-length in Cass County (*n* = 412).

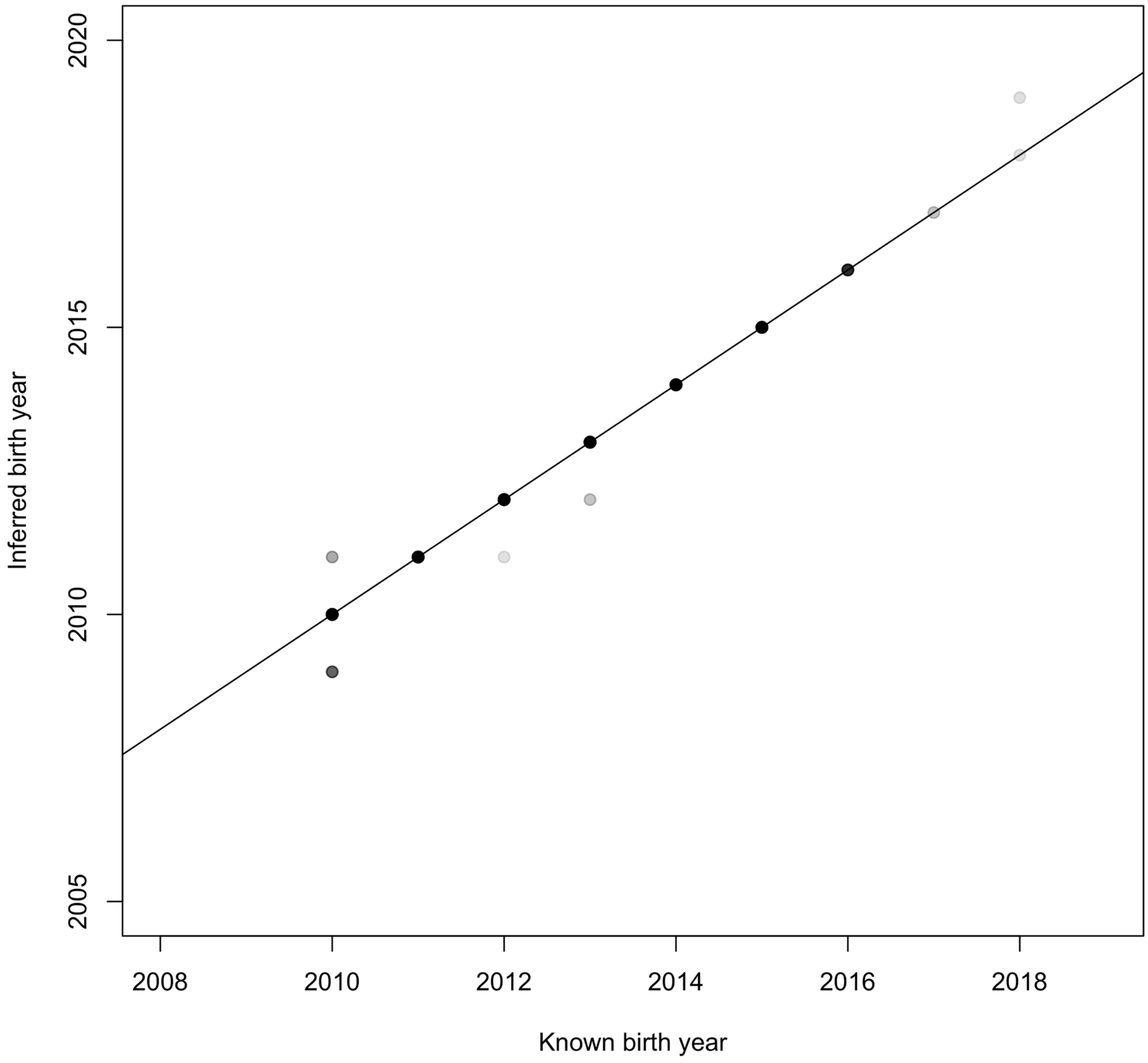

Figure S9. Plot shows the correlation between known individual birth year and birth years inferred using snout-vent-length (SVL) and von Bertalanffy models.

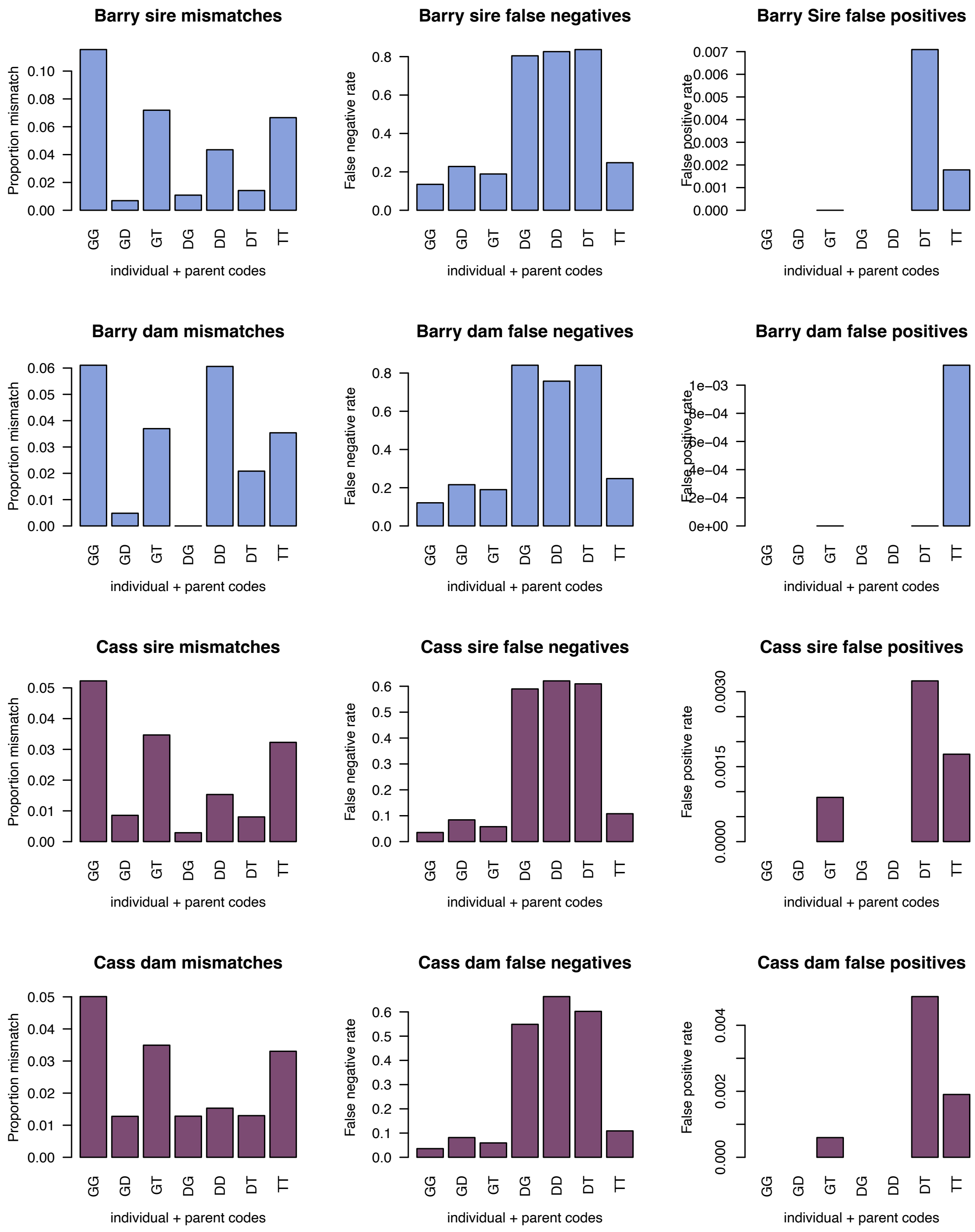

Figure S10. Pedigree errors between the empirical pedigree and pedigrees reconstructed using genotype data simulated from the empirical pedigree (N = 10 simulated pedigrees). Mismatches represent cases when a different parent was assigned to an individual in a simulated versus empirical pedigree. The false negative rate is the rate at which a parent was assigned in the empirical pedigree but was not in the simulated pedigree. The false positive (rate is the rate at which a parent was assigned in the simulated pedigree, but not assigned in the empirical pedigree.

Table S1. Confidence probabilities for different categories of parent-offspring trios in the Barry County pedigree estimated by reconstructing pedigrees from genotype data simulated from the empirical reconstructed pedigree (*n* = 10 simulated pedigrees). Individuals of type “G” are genotyped, “D” are dummy individuals inferred to exist through pedigree relationships, and “X” indicates no individual assigned. Bolded values represent types of pedigree relationships that were used in analyses. Italicized values represent types of relationships that were dropped form analyses due to low confidence.

Focal type	Dam type	Sire time	Dam confidence probability	Sire. confidence probability	Pair confidence probability	N
G	**G**	**G**	**1.000**	**1.000**	1.000	406
G	**G**	D	**1.000**	0.982	0.982	221
G	**G**	X	**0.992**	NA	NA	124
G	D	**G**	0.991	1.000	0.991	213
G	D	D	0.804	0.668	0.585	316
G	D	X	1.000	NA	NA	13
G	X	**G**	NA	**1.000**	NA	69
G	X	D	NA	0.875	NA	16
G	X	X	NA	NA	NA	1162
D	**G**	**G**	**1.000**	**1.000**	1.000	10
D	**G**	D	**1.000**	1.000	1.000	3
D	**G**	X	*0.167*	NA	NA	6
D	D	**G**	0.833	1.000	0.833	6
D	D	D	NA	NA	NA	0
D	D	X	NA	NA	NA	0
D	X	**G**	NA	0.167	NA	6
D	X	D	NA	NA	NA	0
D	X	X	NA	NA	NA	307

Table S2. Confidence probabilities for different categories of parent-offspring trios in the Cass County pedigree estimated by reconstructing pedigrees from genotype data simulated from the empirical reconstructed pedigree (*n* = 10 simulated pedigrees). Individuals of type “G” are genotyped, “D” are dummy individuals inferred to exist through pedigree relationships, and “X” indicates no individual assigned. Bolded values represent types of pedigree relationships that were used in analyses. Italicized values represent types of relationships that were dropped form analyses due to low confidence.

Focal type	Dam type	Sire time	Dam confidence probability	Sire. confidence probability	Pair confidence probability	N
G	**G**	**G**	**1.000**	**1.000**	1.000	2223
G	**G**	D	**0.999**	0.999	0.998	1321
G	**G**	X	**0.991**	NA	NA	108
G	D	**G**	0.993	**0.998**	0.993	1339
G	D	D	0.817	0.804	0.697	1273
G	D	X	0.833	NA	NA	36
G	X	**G**	NA	**0.986**	NA	143
G	X	D	NA	0.953	NA	85
G	X	X	NA	NA	NA	1142
D	**G**	**G**	**0.978**	**0.978**	0.978	89
D	**G**	D	**1.000**	0.952	0.952	62
D	**G**	X	*0.629*	NA	NA	35
D	D	**G**	0.976	1.000	0.975	41
D	D	D	0.875	1.000	0.875	24
D	D	X	1.000	NA	NA	2
D	X	**G**	NA	*0.591*	NA	22
D	X	D	NA	1	NA	10
D	X	X	NA	NA	NA	693

Table S3. Candidate set of von Bertalanffy models (Fabens 1965) constructed from capture-recapture data collected from eastern massasauga populations in Cass and Barry counties between 2009–2023. Model parameters include asymptotic size ($L\infty$) and the growth constant (*K*). ΔAIC_c is the difference between the AIC_c of a given model and the top-ranked model; ω_i is the probability that the model is the most parsimonious given the data and relative to other models in the candidate set; k is the number of parameters in the model; -2(log)$\mathcal{L}$ is the relative fit of the model.

Models	ΔAIC_c	ω_i	k	-2(log)$\mathcal{L}$
L∞(site+sex), *K*(.)	0	0.283	5	-1126.1
L∞(site+sex), *K*(sex)	0.97	0.174	6	-1125.5
L∞(site*sex), K(.)	1.04	0.169	6	-1125.6
L∞(site*sex), K(sex)	2.31	0.089	7	-1125.2
L∞(sex), K(site+sex)	2.66	0.075	6	-1126.4
L∞(sex), K(.)	2.93	0.065	4	-1128.6
L∞(site+sex), K(site+sex)	2.94	0.065	7	-1125.5
L∞(sex), K(sex)	4.04	0.038	5	-1128.1
L∞(site*sex), K(site+sex)	4.39	0.032	8	-1125.2
L∞(site*sex), K(site*sex)	6.48	0.011	9	-1125.2

SI References

1. E. T. Hileman, R. B. King, L. J. Faust, Eastern massasauga demography and extinction risk under prescribed-fire scenarios. *J. Wildl. Manag.* **82**, 977–990 (2018).

2. W. H. Schaefer, Diagnosis of sex in snakes. *Copeia* **1934**, 181 (1934).

3. H. Quinn, J. P. Jones, Squeeze box technique for measuring snakes. *Herpetol. Rev.* **5**, 35 (1974).

4. E. T. Hileman, D. R. Bradke, D. M. Delaney, R. B. King, Protection by association: Implications of scent trailing in neonate eastern massasaugas (*Sistrurus catenatus*). *Herpetol. Conserv. Biol.* (2015).

5. A. L. Stedman, *et al.*, Multiple paternity in three wild populations of eastern massasauga (*Sistrurus catenatus*). *Herpetol. Conserv. Biol.* **11**, 160–167 (2016).

6. J. Catchen, P. A. Hohenlohe, S. Bassham, A. Amores, W. A. Cresko, Stacks: An analysis tool set for population genomics. *Mol. Ecol.* **22**, 3124–3140 (2013).

7. D. A. R. Eaton, PyRAD: assembly of de novo RADseq loci for phylogenetic analyses. *Bioinformatics* **30**, 1844–1849 (2014).

8. S. Mathur, A. J. Mason, G. S. Bradburd, H. L. Gibbs, Functional genomic diversity is correlated with neutral genomic diversity in populations of an endangered rattlesnake. *Proc. Natl. Acad. Sci.* **120**, e2303043120 (2023).

9. O. A. Ali, *et al.*, RAD capture (rapture): Flexible and efficient sequence-based genotyping. *Genetics* **202**, 389–400 (2016).

10. H. Li, Aligning sequence reads, clone sequences and assembly contigs with BWA-MEM. *ArXiv Prepr.* **00**, 1–3 (2013).

11. H. Li, *et al.*, The Sequence Alignment/Map format and SAMtools. *Bioinformatics* **25**, 2078–2079 (2009).

12. H. Li, A statistical framework for SNP calling, mutation discovery, association mapping and population genetical parameter estimation from sequencing data. *Bioinformatics* **27**, 2987–2993 (2011).

13. J. Graffelman, Exploring Diallelic Genetic Markers: The HardyWeinberg Package. *J. Stat. Softw.* **64**, 1–23 (2015).

14. R. C. Team, R: A language and environment for statistical computing. (2016). Deposited 2016.

15. F. I. Archer, P. E. Adams, B. B. Schneiders, stratag: An r package for manipulating, summarizing and analysing population genetic data. *Mol. Ecol. Resour.* **17**, 5–11 (2017).

16. R. K. Waples, W. A. Larson, R. S. Waples, Estimating contemporary effective population size in non-model species using linkage disequilibrium across thousands of loci. *Heredity* **117**, 233–240 (2016).
